# Gadolinium Chloride Inhibits the Production of Liver Interleukin-27 and Mitigates Liver Injury in the CLP Mouse Model

**DOI:** 10.1155/2021/2605973

**Published:** 2021-01-29

**Authors:** Jing Fan, Miao He, Chuan-Jiang Wang, Mu Zhang

**Affiliations:** ^1^Department of Critical Care Medicine, The First Affiliated Hospital of Chongqing Medical University, No. 1 Youyi Road, Yuzhong District, Chongqing 400016, China; ^2^Chongqing University Cancer Hospital, No. 181 Hanyu Road, Shapingba District, Chongqing 400030, China

## Abstract

**Background:**

Liver macrophages play an important regulatory role in the inflammatory response of liver injury after severe infection. Interleukin- (IL-) 27 is an inflammatory cytokine that plays an important role in diseases caused by bacterial infection. However, the relationship between IL-27 and liver macrophages in liver injury after severe infection is not yet clear.

**Methods:**

A cecal ligation puncture (CLP) model was established in wild-type (WT) and IL-27 receptor- (WSX-1-) deficient (IL-27r^−/−^) mice, and recombinant IL-27 and gadolinium chloride (GdCl3) were injected into WT mice in the designated groups. The serum and liver IL-27, IL-6, tumor necrosis factor alpha (TNF-*α*), and IL-1*β* expression levels were evaluated by ELISA, quantitative PCR, or Western blotting; serum ALT and AST were detected by detection kits; and the severity of liver damage was evaluated by hematoxylin and eosin staining and the TUNEL assay of the liver tissue from the different groups. Liver macrophage polarization was evaluated by immunofluorescence. In addition, the polarization of peritoneal macrophage was evaluated by flow cytometry.

**Results:**

The serum and liver IL-27 expression levels were elevated in WT mice after CLP-induced severe infection, which were consistent with the changes in HE scores in the liver tissue. The levels of serum ALT, AST, liver IL-6, TNF-*α*, and IL-1*β* mRNA and liver pathological injury scores were further increased when pretreated with recombinant IL-27 in WT mice, but these levels were decreased in IL-27r^−/−^ mice after CLP-induced severe infection compared to WT mice. In WT mice pretreated with GdCl3, liver pathological scores, serum ALT and AST, TUNEL-positive cell proportion from liver tissues, liver IL-27 expression, and the liver macrophages M1 polarization proportion decreased after CLP; however, the serum IL-27, IL-6, TNF-*α*, and IL-1*β* levels and the pathological lung and kidney scores were not significantly changed. When supplemented with exogenous IL-27, the liver pathological scores, serum ALT, AST, TUNEL-positive cell proportion of liver tissues, liver IL-27 expression, and the liver macrophage M1 polarization proportion increased. The in vitro, IL-27 expression increased in peritoneal macrophages when stimulated with LPS. Recombinant IL-27 together with LPS promoted the elevations in IL-6, TNF-*α*, and IL-1*β* levels in supernatant and the M1 polarization of peritoneal macrophages.

**Conclusion:**

IL-27 is an important cytokine in the inflammatory response to liver injury after severe infection. The reduction of liver injury by gadolinium chloride in severe infection mice models may relate to the inhibition of liver IL-27 production. These changes may be mainly related to the decrease of liver macrophages M1 polarization. IL-27 may have a positive feedback on these macrophages.

## 1. Introduction

Acute liver dysfunction is a concomitant manifestation of many critical illnesses.

Liver dysfunction is sometimes closely related to poor prognosis in critical patients [1, 2]. Severe infection is one of the most important causes of liver dysfunction in critical patients [3]. In severe infection, the liver plays crucial roles in pathogen defense, clearance, and mediating inflammatory responses in the pathophysiology process [4]. However, this involvement is a double-edged sword: the liver removes bacteria and toxins and can also cause inflammation, immune suppression, and organ damage due to an overwhelming systemic inflammatory storm [5]. Interleukin- (IL-) 27 is a heterodimeric cytokine of the IL-12 family composed of the Epstein-Barr virus- (EBV-) induced gene 3 (EBI3) and the p28 subunit and plays an important regulatory role in the inflammatory responses of infectious disease [6, 7]. IL-27 is a biphasic regulator, acting as a pro- or anti-inflammatory factor under different conditions. It is involved in the induction of IL-10 production, Foxp32, and T-regulatory 1 (Tr1) cells and plays an anti-inflammatory effect [8, 9]; in contrast, it can act as a promotor in peritonitis and in ConA-induced liver injury animal models [10, 11].

Liver macrophages, especially Kupffer cells (KCs), play an important role in the regulation of inflammatory after severe infection [12]. In severe infection, activated Kupffer cells can release large amounts of cytokines, chemokines, prostaglandins, leukotrienes, and complement factors, playing an important role in regulating liver and systemic inflammation and promoting liver damage [13–15]. As IL-27 is mainly produced by macrophages and DCs [16], we speculate that macrophages may regulate liver damage by producing IL-27 in severe infections.

Gadolinium chloride (GdCl3) is an inhibitor of Kupffer cells [17]. It has been reported that GdCl3 contributes to the reduction of liver damage by inhibiting the activation of KCs according to many types of liver injury, including ethanol, dimethylnitrosamine, carbon tetrachloride administration (CCl4), and cadmium-induced liver injury [18, 19]. GdCl3 treatment reduced the proinflammatory cytokine levels of liver, including IL-6, TNF-*α*, IL-1*β*, and alleviated liver injury after CLP [20]. However, the exact mechanisms involved in the reduction of liver injury after GdCl3 treatment are not yet clearly known. Therefore, the aim of this study is to investigate whether GdCl3 can reduce liver injury by affecting IL-27 production.

## 2. Materials and Methods

### 2.1. Animal Models

Male C57BL/6 J mice aged 8–10 weeks were used to establish the cecal ligation puncture (CLP) model [21]. The sham group mice have underwent exactly the same procedures as the CLP group, without the cecum ligation and puncture. Wild-type (WT) C57BL/6 J mice were obtained from the Experimental Animal Center of Chongqing Medical University, and IL-27r^−/−^ (WSX-1 knockout) C57BL/6 J mice were purchased from the Jackson Laboratory (Bar Harbor, ME, USA). All the mice were raised under the specific pathogen-free conditions. The animal experiments were approved by the Institutional Animal Care and Use Committee of Chongqing Medical University.

### 2.2. GdCl3 and Recombinant IL-27 Treatment

In the animal experiments, mice were intravenously injected (via tail vein) with GdCl3 (20 mg/kg body weight, Sigma-Aldrich) 24 h before CLP and then injected intraperitoneally with recombinant mouse IL-27 [7] (1 *μ*g; ProSpec-Tany TechnoGene Ltd, Ness-Ziona, Israel) 2 h before CLP; all the groups were observed for 24 h after CLP injection. In the cell experiments, peritoneal macrophages were treated with recombinant IL-27 (50 ng/mL) 2 h before the addition of lipopolysaccharide (LPS, 100 ng/mL; Sigma–Aldrich, St. Louis, MO, USA), followed by incubation for 12 h.

### 2.3. Cell Extraction and Culture

Intraperitoneal injection of 3% thioglycollate is being used in C57BL/6 J mouse for 3 days, then the peritoneal cavity of each mouse was lavaged with 20 mL of PBS. After that, three to five milliliters of abdominal cavity liquid was extracted with a syringe and injected into a sterile centrifuge tube, which was then centrifuged at 1000 r/min for 5 min. The collected macrophages were resuspended in the medium (RPMI-1640 with 10% fetal bovine serum, 100 U/mL penicillin, and 10 *μ*g/mL streptomycin) and then incubated in a 24-well plate at the concentration of 1 × 10^5^ cells/ml with the same medium, in the incubator at 37°C in a humidified atmosphere containing 5% CO_2_. After 2 h of incubation, warm PBS was used to wash the plate, nonadherent cells were removed with PBS, and then the adherent cells were incubated in the same medium and cultivation environment as before. The total liver macrophages of mice in the WT-CLP group and WT-CLP with GdCl3 pretreatment group were extracted as described [22].

### 2.4. HE and TUNEL

The liver, lung, and kidney tissues of mice obtained from each group at the indicated time point were fixed in paraformaldehyde, embedded in paraffin, and stained with hematoxylin and eosin (H&E) or using a terminal deoxynucleotidyl transferase-mediated dUTP nick end labeling (TUNEL) assay kit (apoptosis detection kit (Roche, Mannheim, Germany)) to evaluate the cell apoptosis. Every tissue section was analyzed by light microscopy under ×200 and ×400 magnifications, and dark brown stained cell is positive. The liver, lung, and kidney pathology scoring methods used to evaluate the degree of tissue damage were previously described [23–25]. The degree of apoptosis was quantified based on the proportion of TUNEL-positive cells, counted under ×400 magnification of the microscope, 5 fields of view were taken for each tissue section, and 200 cells were counted in each field of view; the proportion of positive cells was calculated, and the final result of each tissue section is the average of the positive cell proportion of 5 fields of view.

### 2.5. Quantitative Polymerase Chain Reaction Assay

We followed the methods of Dr. Fan et al. 2019 [26]. Quantitative PCR (qPCR) for EBI3, P28, IL-6, TNF-*α*, and IL-1*β* was performed following the protocol by GeneCopoeia, Rockville, MD, USA. Total RNA was extracted from the liver tissue or cells using TRIzol reagent (Invitrogen, Carlsbad, CA, USA) and reverse transcribed into cDNA using a high-capacity cDNA reverse transcription kit (Takara, Tokyo, Japan). The primer sequences were designed and chemically synthesized by Sangon Bio (Shanghai, China): EBI3 forward 5′-GCT GCT CTT CCT GTC ACT TGC C-3′ and reverse 5′-TGA AGG ACG TGG ATC TGG TGG AG-3′; p28 forward 5′-CTG CTT CCT CGC TAC CAC ACT TC-3′ and reverse 5′-CTC TTC CTC CTT GTC CTC CTC CTC-3′ (antisense); TNF-*α* forward 5′-GCG ACG TGG AAC TGG CAG AAG-3′ and reverse 5′-GCC ACA AGC AGG AAT GAG AAG AGG-3′; IL-6 forward 5′-ACT TCC ATC CAG TTG CCT TCT TGG-3′ and reverse 5′-TTA AGC CTC CGA CTT GTG AAG TGG-3′; IL-1*β* forward 5′-TCG CAG CAG CAC ATC AAC AAG AG-3′ and reverse 5′-TGC TCA TGT CCT CAT CCT GGA AGG-3′; glyceraldehyde 3-phosphate dehydrogenase (GAPDH) forward 5′-AGC GAG ACC CCA CTA ACA-3′ and reverse 5′-GGG GCT AAG CAG TTG GTG-3′.

### 2.6. Western Blot Analysis

Following the methods of Dr. Fan et al. 2019 [26], protein samples were harvested from the liver tissue or cells by lysis buffer containing protease and phosphorylation inhibitors, and the protein concentration was determined using a BCA Protein Assay Kit (Beyotime Biotechnology, Shanghai, China). The protein samples were separated by 10% SDS-PAGE and electrotransferred to polyvinylidene fluoride membranes (Millipore, Burlington, MA, USA). The membranes were blocked in QuickBlock™ Blocking Buffer for Western blotting (Beyotime Biotechnology) and then incubated with the following primary antibodies at 4°C overnight: rabbit polyclonal anti-IL-27-A antibody (p28) (1 : 500; Abcam, Cambridge, UK), rabbit polyclonal anti-IL-27-B (EBI3) antibody (1 : 500; AbKlean, Sangon Biotech, Shanghai, China), *β*-actin monoclonal antibody (1 : 1000), and *β*-tubulin monoclonal antibody (1 : 1000) (Boster Biological Technology, Wuhan, China); *β*-actin and *β*-tubulin were used as the sample loading controls. The next day, the membrane was incubated for 1 h at room temperature with peroxidase-conjugated goat anti-rabbit IgG (1 : 5000; ZSGB-BIO, Beijing, China) or goat anti-mouse IgG (1 : 1000; Boster Biological Technology, Wuhan, China) secondary antibody. Enhanced chemiluminescence was used to detect the proteins using a Chemiluminescent Detection Kit (Advansta, San Jose, CA, USA).

### 2.7. Cytokine and Liver Enzymes Assay

Following the methods of Dr. Fan et al. 2019 [26], IL-27 levels (R&D Systems, Minneapolis, MN, USA) in mouse serum and IL-6 (Boster Biological Technology, Wuhan, China) and TNF-*α* and IL-1*β*(R&D Systems) levels in cell supernatant were detected by enzyme-linked immunosorbent assay (ELISA) kits according to the manufacturer's protocol.

Alanine aminotransferase (ALT) and aspartate transaminase (AST) in serum were measured at 24 h after CLP and normal controls using detection kits (Nanjing JianCheng Bioengineering Institute, Jiangsu, China).

### 2.8. Immunofluorescence

The immunofluorescence method for detecting liver macrophages is as follows: first, frozen liver sections were recovered by PBS and then 0.3% Triton X-100 (50 *μ*L) was applied for 15 minutes to rupture the cell membrane. Later, we used sodium citrate solution and heating for antigen retrieval and normal goat serum to block the section. After that, rabbit anti-mouse F4/80 polyclonal antibody was added to the section, and incubation was performed in a humid chamber at 4°C overnight. The next day, the section was kept for 1 h at room temperature, washed in PBS, and then the goat anti-rabbit PE-Cy3 secondary antibody was added for 1 h at room temperature. After that, the section was washed in TBST for 3 times and used sodium citrate solution and heating for antigen retrieval and normal goat serum to block the section again. And then, rabbit anti-mouse CD206 polyclonal antibody was added to the section, incubated in a humid chamber at 4°C overnight. The following day, goat anti-rabbit FITC secondary antibody was added, and the detailed steps were the same as before. After that, the section was washed in TBST for 3 times again and used sodium citrate solution and heating for antigen retrieval and normal goat serum to block the section one more time. Later, rabbit anti-mouse iNOS polyclonal antibody was added to the section, and incubation was performed in a humid chamber at 4°C overnight. The following day, the goat anti-rabbit PE-Cy5 secondary antibody was added, and the detailed steps were the same as before. After washed, the section was added with autofluorescence quencher for 5 minutes and washed with running water for 10 minutes. Then, 4′,6-diamidino-2-phenylindole (DAPI) was used to dye the cell nuclei; after that, the section was washed for 3 times, and antifade mounting medium was used for sealing. Positive cells were counted and analyzed by fluorescence microscope under ×200 magnifications of the microscope, 5 fields of view were taken for each tissue section, the total macrophage numbers, the M1 type macrophages, and the M2 type macrophages numbers were counted separately, the proportion of M1 and M2 type macrophages were calculated, and the final result of each tissue section is the average of the M1 and M2 type macrophage proportion of 5 fields of view. The primary and secondary antibodies were purchased from Servicebio, Wuhan, China.

The immunofluorescence method has also been used to detect the expression of IL-27p28 in liver macrophages. The detailed steps were the same as before. Rabbit anti-mouse F4/80 polyclonal antibody and goat anti-rabbit CY3-TSA secondary antibody were used for identifying the macrophages. And rabbit polyclonal IL-27p28 antibody and goat anti-rabbit FITC secondary antibody were used for detecting the expression of IL-27p28. The expression of IL-27p28 in F4/80-positive cells was analyzed by confocal microscopy under ×200 magnifications of the microscope, and 5 fields of view were taken for each tissue section. Total F4/80-positive cells, both F4/80 and IL-27p28-positive cells, were counted in each view, and the proportion of IL-27p28-positive cells in total F4/80-positive cells wascalculated. The average result of the 5 fields of view is the final result of each tissue section.

### 2.9. Flow Cytometry

The membrane surface molecules of cells were stained with 3 *μ*L/test of PE-conjugated anti-mouse CD86 MAb (Invitrogen, California, USA) or APC-conjugated anti-mouse CD206 MAb (Invitrogen, California, USA) for 30 min at room temperature in the dark as per the manufacturer's instructions. The mean fluorescence intensity (MFI) of the cell surface molecules was assessed by flow cytometry (FCM).

### 2.10. Statistical Analysis

The data in this study are expressed as the means ± standard deviations (SDs), and the statistical significance of differences between two groups was statistically analyzed with the independent *t-*test. The one-way ANOVA or two-way ANOVA analysis was used when three or more groups were being compared. All of the analysis were performed with SPSS 23.0 statistical software and GraphPad Prism 7.04. *p* < 0.05 was considered to be statistically significant.

## 3. Results

### 3.1. IL-27 Expression Is Upregulated in Liver Damage Mice after CLP

**Figure 1:**
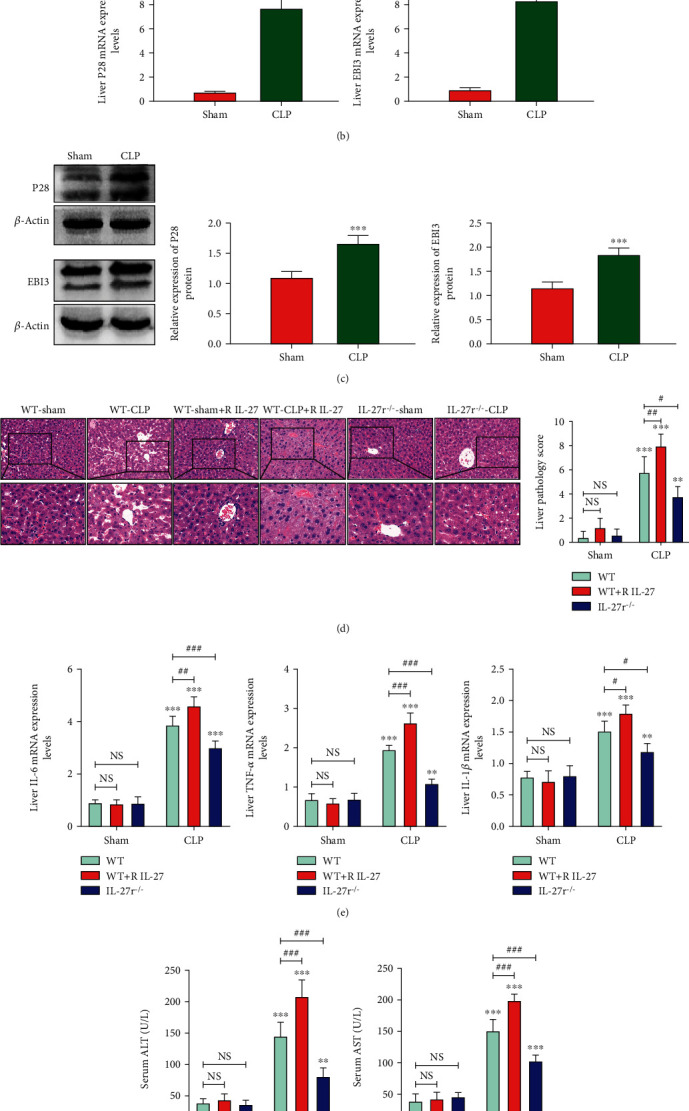
(a) Serum IL-27 levels of sham WT mice (Sham) and WT mice after CLP (CLP) (by ELISA), ^∗∗∗^*p* < 0.001. (b) EBI3 and P28 mRNA levels in the liver tissue of sham WT mice (Sham) and WT mice after CLP (CLP) (by qPCR), ^∗∗∗^*p* < 0.001. (c) EBI3 and P28 protein levels in the liver tissue of sham WT mice (Sham) and WT mice after CLP (CLP) (by Western blot), ^∗∗∗^*p* < 0.001. (d) Hematoxylin and eosin- (H&E-) stained liver tissues and histological scores for the liver from WT, WT+recombinant IL-27 (R IL-27) pretreatment, and IL-27r^−/−^ mice (*n* = 5 per group) (sham and after CLP) (×200, ×400 magnifications), ^∗∗^*p* < 0.01, ^∗∗∗^*p* < 0.001 vs sham group; NS: *p* > 0.05, #*p* < 0.05, ##*p* < 0.01. (e) IL-6, TNF-*α*, and IL-1*β* mRNA levels in the liver tissue (by qPCR) of WT, WT+recombinant IL-27 (R IL-27) pretreatment, and IL-27r^−/−^ mice (sham and after CLP), ^∗∗^*p* < 0.01, ^∗∗∗^*p* < 0.001 vs sham group; NS: *p* > 0.05, #*p* < 0.05, ##*p* < 0.01, ###*p* < 0.001. (f) Serum ALT and AST levels of WT, WT+recombinant IL-27 (R IL-27) pretreatment, and IL-27r^−/−^ mice (sham and after CLP). ^∗∗^*p* < 0.01, ^∗∗∗^*p* < 0.001 vs sham group; NS: *p* > 0.05, ###*p* < 0.001.

To verify the relationship between IL-27 and liver injury in a CLP-induced severe infection mouse model, we first detected IL-27 levels in serum by ELISA and the expression levels of EBI3 and P28 (subunits of IL-27) in the liver tissue by q-PCR and Western blot. We found that, compared to the sham group, the serum level of IL-27 was increased after CLP (*p* < 0.001; Figure 1(a)), and the liver EBI3 and P28 mRNA (by q-PCR) and protein expression levels (by Western blot) were also upregulated after CLP (*p* < 0.001; Figures 1(b) and 1(c)). Meanwhile, the levels of the inflammatory factors IL-6, TNF-*α*, and IL-1*β* in the liver tissue, determined by q-PCR, were also increased at the same time point after CLP (*p* < 0.001; Figure 1(e)). The histological scores of liver injury and serum ALT and AST levels also increased under the same conditions (*p* < 0.001; Figures 1(d) and 1(f)).

### 3.2. IL-27 Aggravates Liver Injury after CLP

Next, we used IL-27r^−/−^ (WSX-1 deficient) mice and intraperitoneal injection of recombinant IL-27 to further verify the effect of IL-27 on liver injury after CLP. The histological scores of liver injury and serum ALT and AST levels increased in the WT mice group after CLP with recombinant IL-27 treatment and decreased in IL-27r^−/−^ mice group after CLP (*p* < 0.05, *p* < 0.01, *p* < 0.001; Figures 1(d) and 1(f)). The IL-6, TNF-*α*, and IL-1*β* mRNA levels of the liver tissue had the same changes according to these groups (*p* < 0.05, *p* < 0.01, *p* < 0.001; Figure 1(e)). Our results indicated that IL-27 plays an import role in aggravating liver injury after CLP.

### 3.3. GdCl3 Pretreatment Attenuates Liver Injury and Liver Cell Apoptosis after CLP

**Figure 2:**
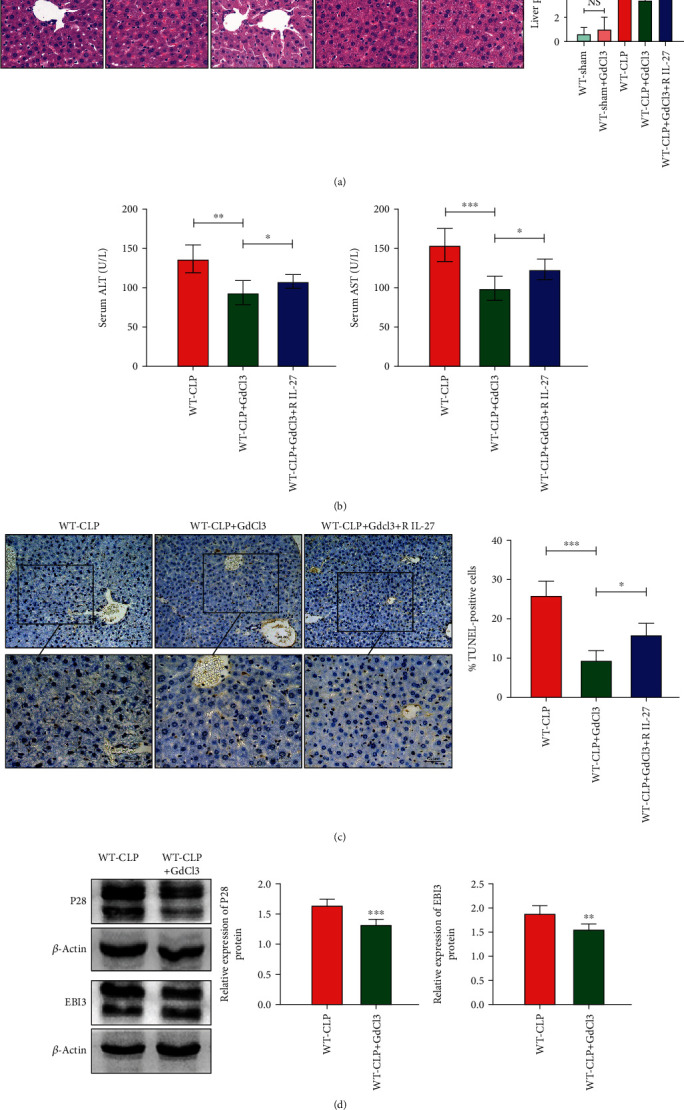
(a) Hematoxylin and eosin- (H&E-) stained liver tissues and histological scores for liver from WT mice (*n* = 5 per group) with or without GdCl3 and recombinant IL-27 (R IL-27) pretreatment (sham and after CLP), ^∗^p < 0.05, ^∗∗^*p* < 0.01, ^∗∗∗^*p* < 0.001, NS: *p* > 0.05. (b) Serum ALT and AST levels of WT mice after CLP with or without GdCl3 and recombinant IL-27 (R IL-27) pretreatment, ^∗^*p* < 0.05, ^∗∗^*p* < 0.01, ^∗∗∗^*p* < 0.001. (c) DNA fragmentation analysis (TUNEL) and TUNEL-positive cell proportion of the liver from WT mice (*n* = 5 per group) after CLP with or without GdCl3 and recombinant IL-27 (R IL-27) pretreatment (×200, ×400 magnifications), ^∗^*p* < 0.05, ^∗∗^*p* < 0.01. (d) EBI3 and P28 protein levels in the liver tissue of WT mice after CLP with or without GdCl3 pretreatment. ^∗∗^*p* < 0.01, ^∗∗∗^*p* < 0.001.

Next, we verified whether GdCl3 pretreatment can reduce liver damage or liver cell apoptosis after CLP. The liver pathological scores and serum ALT and AST levels decreased in the CLP mouse group with GdCl3 pretreatment (*p* < 0.01, *p* < 0.001; Figures 2(a) and 2(b)). We also found that the TUNEL-positive cell proportion in the liver decreased in the CLP mouse group with GdCl3 pretreatment (*p* < 0.01; Figure 2(c)) when compared to the CLP mouse group without GdCl3 pretreatment. When treated with recombinant IL-27, the pathological scores of liver and serum ALT and AST levels after CLP in the GdCl3 pretreatment groups increased (*p* < 0.05; Figures 2(a) and 2(b)) but were still lower than those of the CLP groups without GdCl3 pretreatment. We also found the same changes in TUNEL-positive cell proportion under the same conditions (*p* < 0.05; Figure 2(c)).

### 3.4. GdCl3 Pretreatment Is Involved in the Reduction of IL-27 in the Liver after CLP

To explore whether the reduction of liver injury by GdCl3 pretreatment in the CLP mouse model is related to the IL-27 level, we detected the IL-27 expression in the liver after CLP when pretreated with GdCl3. Western blot showed that the expression levels of the EBI3 and P28 subunits decreased after CLP when mice were pretreated with GdCl3, compared to the group without GdCl3 pretreatment (*p* < 0.01, *p* < 0.001; Figure 2(d)).

### 3.5. GdCl3 Pretreatment Does Not Significantly Reduce the Levels of Serum IL-27 and Circulatory Proinflammatory Factors or the Pathological Damage to Other Organs after CLP

**Figure 3:**
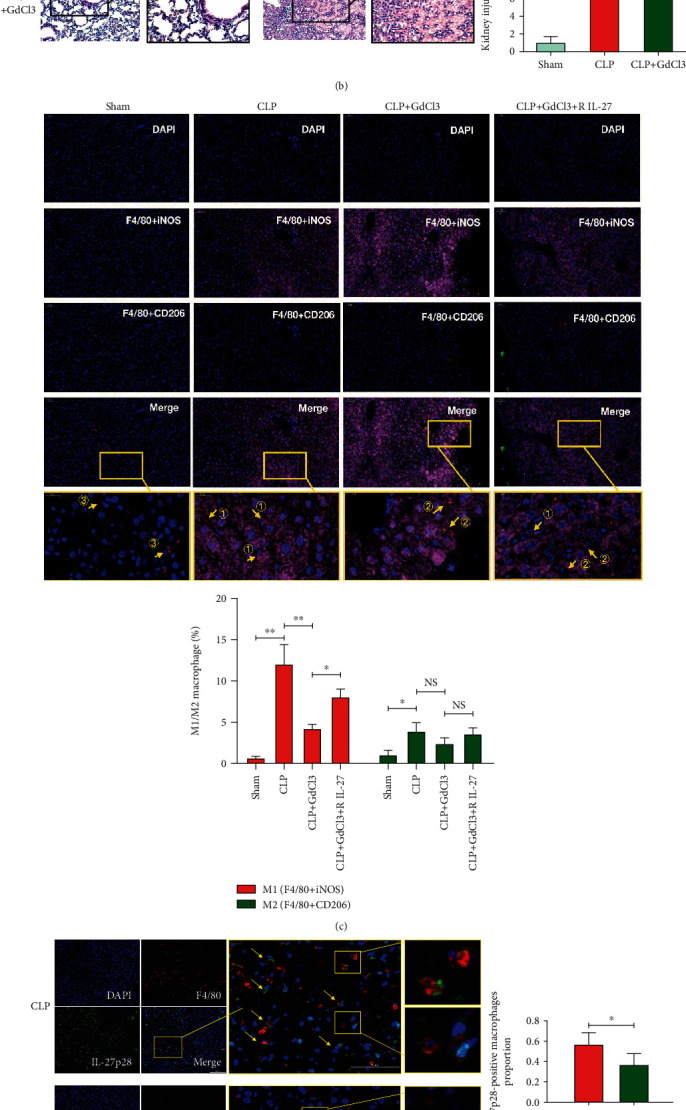
(a) Serum IL-27, IL-6, TNF-*α*, and IL-1*β* levels (by ELISA) of WT mice after CLP with or without GdCl3 pretreatment, NS:*p* > 0.05. (b) Hematoxylin and eosin- (H&E-) stained lung and kidney tissues and histological scores from sham WT mice (Sham) and WT mice after CLP (*n* = 5 per group) with or without GdCl3 pretreatment (×200, ×400 magnifications), ^∗∗^*p* < 0.01, NS: *p* > 0.05. (c) M1 and M2 polarization numbers of liver macrophages in sham WT mice (sham) and WT mice after CLP (*n* = 5 per group) with or without GdCl3 and recombinant IL-27 (R IL-27) pretreatment (by immunofluorescence) (×200, ×800 magnifications). Blue fluorescence stands for DAPI, red fluorescence stands for F4/80, green fluorescence stands for CD206, and pink fluorescence stands for iNOS. The F4/80 + iNOS image showed only the M1 type macrophages (cells with red and pink color), while the F4/80 + CD206 image showed only the M2 type macrophages (cells with red and green color), and the merged image showed both the M1 type and M2 type of macrophage. ① stands for M1 type macrophages, ② stands for M2 type macrophages, and ③ stands for macrophages that are not polarized to M1 or M2 type. ^∗^*p* < 0.05, ^∗∗^*p* < 0.01, NS: *p* > 0.05. (d) The IL-27p28 expression in liver macrophages after CLP (*n* = 5 per group) with or without GdCl3 pretreatment (by immunofluorescence) (confocal microscopy ×200, ×600 magnifications). Blue fluorescence stands for DAPI, red fluorescence stands for F4/80, and green fluorescence stands for IL-27p28. ^∗^*p* < 0.05. (e) EBI3 and P28 mRNA levels of liver macrophages in WT-CLP and WT-CLP + GdCl3 mice groups (by qPCR), ^∗^*p* < 0.05.

We then detected the serum levels of IL-27 and the proinflammatory factors IL-6, TNF-*α*, and IL-1*β* in the CLP mouse groups with or without GdCl3 pretreatment. The results showed that, although the serum levels of IL-27, IL-6, TNF-*α*, and IL-1*β*decreased in the CLP mouse group with GdCl3 pretreatment, the difference was not statistically significant (*p* > 0.05; Figure 3(a)). Additionally, HE staining of the lung and kidney showed that the lung and kidney damages, as calculated by their pathological scores, were not significantly reduced in the CLP mouse group with GdCl3 pretreatment when compared to the CLP mouse group without GdCl3 pretreatment (*p* > 0.05; Figure 3(b)).

### 3.6. GdCl3 Pretreatment Involved in the Reduction of the IL-27 Expression in Mice Liver Macrophages after CLP, which May Be Related to the Changes in the Polarization State of Liver Macrophages

Immunofluorescence revealed that the proportion of iNOS+ macrophages in the mouse liver increased after CLP and decreased when mouse were pretreated with GdCl3 (*p* < 0.01; Figure 3(c)). Moreover, the proportion of CD206+ macrophages in the mouse liver also increased after CLP (*p* < 0.05; Figure 3(c)), but there were not obvious reduction in the proportion of CD206+ macrophages in the livers of CLP mice with GdCl3 pretreatment (*p* > 0.05; Figure 3(c)). When pretreated with recombinant IL-27, the proportion of iNOS+ macrophages in the liver was elevated in CLP mice with GdCl3 pretreatment (*p* < 0.05; Figure 3(c)), while the proportion of CD206+ macrophages exhibited no apparent change under the same condition (*p* > 0.05; Figure 3(c)). Then, we detected the expression of IL-27p28 in liver macrophages by immunofluorescence and found that the expression of IL-27p28 in F4/80+ cells decreased in the WT-CLP + GdCl3 mice group, when compared to the WT-CLP mice group (*p* < 0.001; Figure 3(d)). We also extracted the total macrophages of the mice liver in the WT-CLP and WT-CLP + GdCl3 group, and the qPCR showed that EBI3 and p28 mRNA levels in the WT-CLP + GdCl3 mice group were lower than the WT-CLP mice group (*p* < 0.05; Figure 3(e)). These results indicated that GdCl3 pretreatment was involved in the reduction of the IL-27 expression in the mice liver macrophages after CLP, which may be related to the changes in the polarization state of liver macrophages.

### 3.7. IL-27 Is Elevated in Macrophages and Promotes the Inflammatory Reaction

**Figure 4:**
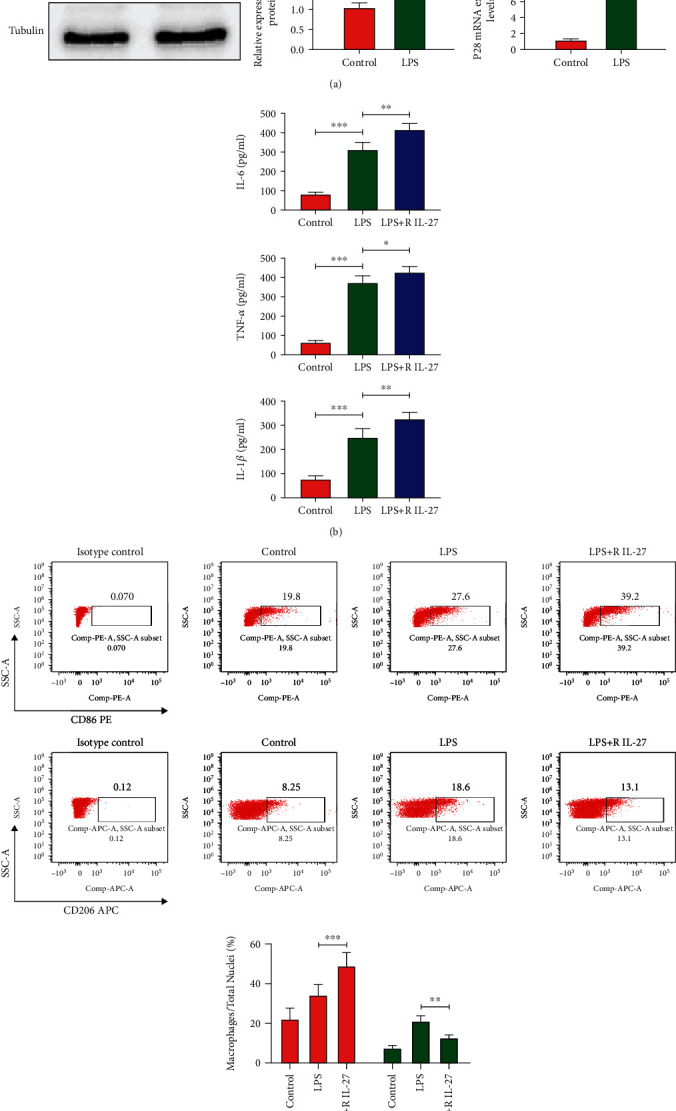
(a) EBI3 and P28 protein and mRNA levels of peritoneal macrophages stimulated by LPS(100 ng/ml) (by western-blot and qPCR), ^∗∗∗^*p* < 0.001 vs control. (b) IL-6, TNF-*α*, and IL-1*β* levels in cell supernatant after LPS stimulated, with or without recombinant IL-27 (R IL-27) (by ELISA), ^∗^*p* < 0.05, ^∗∗^*p* < 0.01, ^∗∗∗^*p* < 0.001. (c) M1 and M2 polarization of peritoneal macrophages after LPS stimulated, with or without recombinant IL-27 (R IL-27) (by flow cytometry). ^∗∗^*p* < 0.01, ^∗∗∗^*p* < 0.001.

To evaluate the IL-27 production of macrophages in an inflammatory state, we detected EBI3 and P28 mRNA levels by q-PCR and protein levels by Western blot of peritoneal macrophages after LPS stimulation. We found that the EBI3 and P28 mRNA and protein expression levels all increased following LPS stimulation (*p* < 0.001; Figure 4(a)). Moreover, when we added recombinant IL-27, the expression levels of the proinflammatory factors IL-6, TNF-*α*, and IL-1*β* in the supernatant were increased (*p* < 0.05, *p* < 0.01; Figure 4(b)). Meanwhile, the flow cytometric analysis showed that the CD86+ macrophages increased after LPS stimulated, which had a further elevation when pretreated with recombinant IL-27 (*p* < 0.001; Figure 4(c)). These results indicated that active peritoneal macrophages can produce IL-27, which may relate to the polarization of macrophages, and played a role in the production of proinflammatory cytokines in vitro, and IL-27 also can affect the macrophages.

## 4. Discussion

Acute hepatic injury is less common than other types of organ dysfunction (e.g., respiratory, renal, and neurological dysfunction) after severe infection [27–30], but it is closely related to the prognosis of patients and is an independent predictor of mortality [31–33]. The pathophysiology of liver dysfunction after severe infection is complex and is not yet well understood [34]. The expanding inflammatory response due to pro- and anti-inflammatory balance disorders caused by infection plays an important role in this pathophysiology. The liver's immune system plays an important role in these processes [35]. Inflammatory factors play an important role in the uncontrolled amplification of inflammatory cascades after liver injury.

As an inflammatory cytokine, IL-27 plays an important role in infection disease, promotes the inflammatory response of sepsis [36], and acts as a marker in predicting bacterial infection in critically ill children [37]. In addition, previous studies have shown IL-27 playing a role in liver injury or failure [11, 38, 39]. In our previous study, we found IL-27 is elevated in sepsis and sepsis-associated liver injury patients and animal models, which is related to the proinflammatory factors and the severity of sepsis, and promoted liver injury [26]. The results of this study confirmed that IL-27 levels in the serum and liver of mice were elevated after CLP-induced bacterial infection, and IL-27 played an important role in promoting inflammatory injury to the liver after CLP.

As IL-27 is mainly produced by macrophages, and the liver has the largest proportion of macrophages among all solid organs of the human body [40], we speculated that macrophages may affect liver injury partly by regulating IL-27 production. As the resident macrophages in the liver, KCs play a vital role in maintaining homeostasis in the liver itself and throughout the body, including removing bacteria and microorganisms that reach the liver and acting as a gatekeeper, initiating, or suppressing the immune response [41]. Activated KCs can produce proinflammatory factors aggravated by liver injury and an inflammatory response [42, 43], and inhibiting KCs can provide a survival advantage in sepsis [44]. Other macrophages in liver including monocyte-derived macrophages, myeloid dendritic cells, inflammatory macrophages, and peritoneal macrophages also play an important role in the inflammatory response of the liver [12, 45]. Therefore, studying on the function of macrophages may be helpful to take a further insight into the mechanism of AHI after severe infection.

GdCl3 is an inhibitor of KCs. Ingestion of gadolinium by phagocytes induces chelation of the gadolinium and phosphate complex, leading to selective depletion and/or inhibition of its antipathogenic and immune-regulatory functions [46–48]. Previous studies have found that GdCl3 can influence the Kupffer cell-related pathophysiological processes of drug-induced liver toxicity, lung inflammation, and ischemia-reperfusion [49–53]. In a CLP-induced severe infection rat model, it was found that the inflammatory factors levels in the serum and liver were decreased when rats were pretreated with GdCl3 [42], while Ravinder et al. found that the serum cytokine and chemokine levels in the GdCl3-pretreated CLP mice group were not different from the CLP mice group without GdCl3-pretreatment [20]. Our results showed that GdCl3 pretreatment attenuated liver injury and liver cell apoptosis after CLP. However, there were no significant changes in serum IL-6, TNF-*α,* and IL-1*β* levels under the same condition; moreover, we did not observe any reductions in damage to the lungs and kidneys (organs that are vulnerable to severe infection) in CdCl3-pretreated mice after CLP. The differences between the studies may be because of the models and the severity of infection. More studies should be performed to explore the specific mechanism of the effect of GdCl3 on AHI.

In this study, we also found that the IL-27 expression is decreased in the liver when pretreated with GdCl3 in a CLP-induced severe infection mouse model. When supplemented with exogenous IL-27, the serum ALT and AST levels, the liver injury scores, and liver cell apoptosis proportion increased. These findings indicated that GdCl3 is involved in reducing IL-27 production in the liver which is related to the reduction of liver damage.

Macrophages are highly heterogeneous cells that can differentiate into alternatively activated macrophages (M1 and M2, respectively), and their population may be critical for protection or promotion of liver injury and diseases when stimulated by bacteria infection and other factors [54–56]. The M1-polarized macrophages produce IL-6, TNF-*α*, and other proinflammatory factors, while M2-polarized macrophages produce IL-10 and other anti-inflammatory factors and play different roles in the inflammatory process [57]. In our study, we found the expression of IL-27p28 decreased in F4/80+ cells of the liver tissue after CLP when pretreated with GdCl3, and the EBI3 and P28 mRNA levels in liver macrophages of mice also decreased under the same condition. We also observed a significant decrease in the iNOS+ macrophage proportion of the liver after CLP with GdCl3 pretreatment. These results indicated that macrophages played an important role in regulating IL-27 levels of the liver in the severe infection mice model, and the decrease in IL-27 levels in the liver macrophages may be related to the inhibition in M1 polarization of macrophages by GdCL3. In a vitro experiment, we confirmed that the peritoneal macrophages can produce IL-27 under LPS stimulation, which may relate to the increase in macrophage M1 polarization.

In addition, the versatility of liver macrophages indicate that some macrophages can replace each other to a certain extent when they are selectively depleted (including Kupffer cells, monocytes), and these cells can adjust its phenotype based on molecular signals from healthy or damaged livers [12]. Therefore, some macrophages in the liver may functionally complement Kupffer cells in the immune regulation process, especially when KCs were depleted [35, 58–60]. Previous studies indicated that IL-27 was involved in regulating the immune activity of macrophages and affected the inflammatory reaction [61, 62]. In the animal experiment in this study, we found that the iNOS+ macrophage proportion increased in the GdCl3-pretreated CLP mouse model when supplemented with exogenous IL-27, which indicated that IL-27 may be also involved in the activity of other macrophages (maybe non-Kupffer cell macrophage populations) in the liver when the KCs were inhibited. These macrophage populations may be also contributing to the inflammation of the liver in the severe infection mouse model. In a vitro experiment, exogenous IL-27 treatment together with LPS promoted the elevation of proinflammatory cytokines in the supernatants of peritoneal macrophages and the proportion of CD86+ peritoneal macrophages. These results indicated that IL-27 may play a role in macrophage polarization to participate in the proinflammatory response and may have a positive feedback on macrophages.

Therefore, we speculated that the liver damage reduced by GdCl3 in the severe infection model may be related to the decrease in liver IL-27 levels, which may be related to the decrease of the M1 polarization proportion of liver macrophages. As the previous studies reported that GdCl3 can inhibit the function of KCs, we speculated that the changes of the IL-27 level in the liver in the severe infection mouth model may be related to the inhibition of KCs. However, the mechanism of GdCl3 targeting inhibition of KCs is still controversial, and more studies are needed to clarify. Therefore, in order to take a deep insight into the role of liver macrophages in the severe infection model, further studies on the directly involvement of KCs and the direct relationship between IL-27 and KCs in this model and its mechanisms will be performed in the future. Also, study on the role of other macrophage population in the liver of this model, detection of changes in liver macrophage subtypes of this model will be performed. In addition, the state of inflammatory cells and the expression of inflammatory factors may be different in different stages of inflammation; so, further studies are needed to explore these mechanisms.

IL-27 plays a role in promoting inflammation in the liver in response to CLP-induced severe infection, but it is not the only cytokine likely to be involved in this process. As a member of the IL-12 family, IL-27 has many similar effects as IL-12, and some of its activities overlap with that of IL-12 [63]. Previous study indicated that IL-12 is an indicator of liver damage, involved in the aggravation of liver injury in a mouse model stimulated by high-dose LPS during the recovery period after CLP [64]. This result suggests that IL-12 plays an important role in liver injury. And based on our findings and the relationship of IL-12 and IL-27, we speculate that both of these cytokines work in conjunction to promote inflammation in the liver in this model, and maybe there would be also a decrease in liver IL-12 production which related to the inhibition of M1 polarization levels of liver macrophages when pretreated with GdCl3 in the CLP model, followed by the reduction of liver damage. Therefore, further studies will be carried out to explore the effect of IL-12 in the same models.

In addition, in the experiment related to the CLP model, the optimal time for injection of recombinant IL-27 is not clear, maybe two hours before the CLP surgery is short, but we observed the difference in the degree of liver injury between the CLP group and sham group following injection of the recombinant IL-27. Therefore, further studies are needed to explore the optimal time for the injection of recombinant IL-27 in CLP models.

## 5. Conclusion

In all, the results of this study showed that IL-27 is an important factor in the uncontrolled inflammatory response in the pathophysiology of liver injury; gadolinium chloride can inhibit IL-27 production in the liver and involved in the reduction of liver injury, which may be mainly related to the decrease in M1 polarization of macrophages, and due to the diversity of liver macrophages, IL-27 may exert a positive feedback on these macrophages.

## Data Availability

The data used to support the findings of this study are available from the corresponding author upon request.
